# Postmenopausal Hormone Therapy and Colorectal Cancer Risk by Molecularly Defined Subtypes and Tumor Location

**DOI:** 10.1093/jncics/pkaa042

**Published:** 2020-05-19

**Authors:** Julia D Labadie, Tabitha A Harrison, Barbara Banbury, Efrat L Amtay, Sonja Bernd, Hermann Brenner, Daniel D Buchanan, Peter T Campbell, Yin Cao, Andrew T Chan, Jenny Chang-Claude, Dallas English, Jane C Figueiredo, Steven J Gallinger, Graham G Giles, Marc J Gunter, Michael Hoffmeister, Li Hsu, Mark A Jenkins, Yi Lin, Roger L Milne, Victor Moreno, Neil Murphy, Shuji Ogino, Amanda I Phipps, Lori C Sakoda, Martha L Slattery, Melissa C Southey, Wei Sun, Stephen N Thibodeau, Bethany Van Guelpen, Syed H Zaidi, Ulrike Peters, Polly A Newcomb

**Affiliations:** p1 Public Health Sciences Division, Fred Hutchinson Cancer Research Center, Seattle, WA, USA; p2 Department of Epidemiology, University of Washington, Seattle, WA, USA; p3 Division of Clinical Epidemiology and Aging Research, German Cancer Research Center (DKFZ), Heidelberg, Germany; p4 Division of Cancer Epidemiology and Genetics, National Cancer Institute, National Institutes of Health, Bethesda, MD, USA; p5 Division of Preventive Oncology, German Cancer Research Center (DKFZ) and National Center for Tumor Diseases (NCT), Heidelberg, Germany; p6 Colorectal Oncogenomics Group, Department of Clinical Pathology, The University of Melbourne, Parkville, Victoria, Australia; p7 Behavioral and Epidemiology Research Group, American Cancer Society, Atlanta, GA, USA; p8 Division of Public Health Sciences, Department of Surgery, Washington University School of Medicine, St Louis, MO, USA; p9 Alvin J. Siteman Cancer Center at Barnes-Jewish Hospital and Washington University School of Medicine, St Louis, MO, USA; p10 Division of Gastroenterology, Department of Medicine, Washington University School of Medicine, St Louis, MO, USA; p11 Division of Gastroenterology, Massachusetts General Hospital, Boston, MA, USA; p12 Clinical and Translational Epidemiology Unit, Department of Medicine, Massachusetts General Hospital, Boston, MA, USA; p13 Division of Cancer Epidemiology, German Cancer Research Center (DKFZ), Heidelberg, Germany; p14 University Medical Centre Hamburg-Eppendorf, University Cancer Centre Hamburg (UCCH), Hamburg, Germany; p15 Cancer Epidemiology Division, Cancer Council Victoria, Melbourne, Victoria, Australia; p16 Centre for Epidemiology and Biostatistics, Melbourne School of Population and Global Health, The University of Melbourne, Melbourne, Victoria, Australia; p17 Department of Medicine, Samuel Oschin Comprehensive Cancer Institute, Cedars-Sinai, Los Angeles, CA, USA; p18 Department of Preventive Medicine, Keck School of Medicine, University of Southern California, Los Angeles, CA, USA; p19 Lunenfeld Tanenbaum Research Institute, Mount Sinai Hospital, University of Toronto, Toronto, Ontario, Canada; p20 Precision Medicine, School of Clinical Sciences at Monash Health, Monash University, Clayton, Victoria, Australia; p21 Nutrition and Metabolism Section, International Agency for Research on Cancer, World Health Organization, Lyon, France; p22 Department of Biostatistics, University of Washington, Seattle, WA, USA; p23 Oncology Data Analytics Program, Catalan Institute of Oncology-IDIBELL, L’Hospitalet de Llobregat, Barcelona, Spain; p24 Program in MPE Molecular Pathological Epidemiology, Department of Pathology, Brigham and Women’s Hospital, Harvard Medical School, Boston, MA, USA; p25 Division of Research, Kaiser Permanente Northern California, Oakland, CA, USA; p26 Department of Internal Medicine, University of Utah, Salt Lake City, UT, USA; p27 Genetic Epidemiology Laboratory, Department of Clinical Pathology, University of Melbourne, Melbourne, Victoria, Australia; p28 Division of Laboratory Genetics, Department of Laboratory Medicine and Pathology, Mayo Clinic, Rochester, MN, USA; p29 Department of Radiation Sciences, Oncology Unit, Umeå University, Umeå, Sweden; Wallenberg Centre for Molecular Medicine, Umeå University, Umeå, Sweden; p30 Ontario Institute for Cancer Research, Toronto, Ontario, Canada

## Abstract

**Background:**

Postmenopausal hormone therapy (HT) is associated with a decreased colorectal cancer (CRC) risk. As CRC is a heterogeneous disease, we evaluated whether the association of HT and CRC differs across etiologically relevant, molecularly defined tumor subtypes and tumor location.

**Methods:**

We pooled data on tumor subtypes (microsatellite instability status, CpG island methylator phenotype status, *BRAF* and *KRAS* mutations, pathway: adenoma-carcinoma, alternate, serrated), tumor location (proximal colon, distal colon, rectum), and HT use among 8220 postmenopausal women (3898 CRC cases and 4322 controls) from 8 observational studies. We used multinomial logistic regression to estimate odds ratios (OR) and 95% confidence intervals (CIs) for the association of ever vs never HT use with each tumor subtype compared with controls. Models were adjusted for study, age, body mass index, smoking status, and CRC family history. All statistical tests were 2-sided.

**Results:**

Among postmenopausal women, ever HT use was associated with a 38% reduction in overall CRC risk (OR =0.62, 95% CI = 0.56 to 0.69). This association was similar according to microsatellite instability, CpG island methylator phenotype and *BRAF* or *KRAS* status. However, the association was attenuated for tumors arising through the serrated pathway (OR = 0.81, 95% CI = 0.66 to 1.01) compared with the adenoma-carcinoma pathway (OR = 0.63, 95% CI = 0.55 to 0.73; *P*_het _=.04) and alternate pathway (OR = 0.61, 95% CI = 0.51 to 0.72). Additionally, proximal colon tumors had a weaker association (OR = 0.71, 95% CI = 0.62 to 0.80) compared with rectal (OR = 0.54, 95% CI = 0.46 to 0.63) and distal colon (OR = 0.57, 95% CI = 0.49 to 0.66; *P*_het _=.01) tumors.

**Conclusions:**

We observed a strong inverse association between HT use and overall CRC risk, which may predominantly reflect a benefit of HT use for tumors arising through the adenoma-carcinoma and alternate pathways as well as distal colon and rectal tumors.

Colorectal cancer (CRC) is a heterogeneous disease that evolves through multiple pathways defined by genetic and epigenetic events (1, 2). Four tumor markers have been commonly used to better characterize this heterogeneity: microsatellite instability (MSI), CpG island methylator phenotype (CIMP), somatic mutations in *BRAF*, and somatic mutations in *KRAS*. Together, these tumor markers approximate 3 distinct molecular pathways of colorectal carcinogenesis: adenoma-carcinoma (traditional), alternate, and serrated (1, 3, 4). These pathways are established early in disease pathogenesis and can be identified within precancerous lesions by microscopy (3, 5–8). Research has shown that these tumor types have distinct appearances, predilections for locations within the colon, and biologic behaviors (8–10). As such, it is plausible that the epidemiologic factors underlying their etiologies could also differ.

Multiple lines of evidence, including randomized controlled trials, show that postmenopausal hormone therapy (HT) is associated with a decreased risk of CRC (11–20). The reduction in risk, about 20%–40% in recent analyses, has been observed in users of estrogen alone as well as combined estrogen plus progestin. Few studies have evaluated whether the association of HT use and CRC risk differs by molecularly defined CRC subtypes; however, such information might increase the understanding of the mechanisms for this beneficial effect. Current literature suggests that HT use is associated with a lower risk of MSI-low or microsatellite stable tumors (MSI-L/MSS) and possibly with a lower risk of CIMP-negative and *BRAF* wild-type tumors (21, 22). HT use has only been associated with *KRAS* wild-type tumors in the distal colon in 1 previous study (23). Regarding tumor location, the association of HT use and CRC is reportedly stronger among tumors of the distal colon compared with the proximal colon (22, 24). To our knowledge, no study has evaluated both tumor markers and location in relation to HT use to provide a comprehensive understanding of subtype-specific CRC risk.

In this study, we examined HT use in relation to molecularly defined CRC subtypes using available data from the Colon Cancer Family Registry (CCFR) (21, 25, 26) and 7 studies contributing to the Genetics of Epidemiology of Colorectal Cancer Consortium (27, 28). Specifically, we evaluated each of the 4 common tumor markers (MSI, CIMP, *BRAF*, and *KRAS*) separately, as well as 3 pathways of carcinogenesis defined by combinations of those markers and tumor location.

## Methods

### Study Populations

Data from 8 observational studies of CRC were pooled: the Cancer Prevention Study II (CPS-II) (29), the German Darmkrebs: Chancen der Verhutung durch Screening Study (DACHS) (30, 31), the Diet Activity and Lifestyle Study (DALS) (32), the Swedish population of the European Prospective Investigation into Cancer (EPIC) (33), the Melbourne Collaborative Cohort Study (MCCS) (34), the Nurses’ Health Study (NHS) (35, 36), the Northern Sweden Health and Disease Study (NSHDS) (37), and 3 population-based sites from the Colon Cancer Family Registry (21, 25, 26). Each study included women diagnosed with incident invasive CRC and contemporaneous unaffected controls. Only women with tumor marker data were eligible for inclusion in this analysis. Study-specific details are described in the Supplementary Methods (available online). All participants provided informed consent for participating in this research, and studies were approved by their respective Institutional Review Boards.

### Data Collection and Harmonization

The harmonization procedure and ascertainment of HT use are described in more detail in the Supplementary Methods (available online). Information on demographics and environmental risk factors was collected via telephone or in-person interviews and/or structured self-completed questionnaires (24, 38, 39). HT use was generally ascertained as any self-reported use at baseline survey. Additionally, ever use of formulation-specific (estrogen-only or estrogen plus progestin) HT use was derived from 3 studies (CCFR, CPSII, and NHS). HT nonusers at reference time were used as the comparison group. Postmenopausal status was harmonized as either 1) study-derived menopausal status, if available; 2) self-reported menopausal status, if study-derived data were not available; or 3) age 55 years and older, if study-derived and self-reported data were not available (40).

### Tumor Characteristics and Molecular Subtyping

Tumor marker testing was conducted using DNA extracted from formalin-fixed, paraffin-embedded tumor tissue specimens. Individual study protocols varied, as outlined below and further detailed in the Supplementary Methods (available online).

MSI testing was primarily conducted using polymerase chain reaction (PCR) following the National Cancer Institute Bethesda Consensus Panel (CCFR, CPS-II, MCCS, NHS) (41). Typically, 4 or more interpretable markers were required to classify tumors, with some variation across studies outlined in Supplementary Table 1 (available online). Additional methods used include immunohistochemistry (NSHDS, EPIC, and a subset of CCFR and MCCS) and mononucleotide marker panels (DACHS, DALS) (Supplementary Methods, available online). Tumors were classified as MSI-high (MSI-H) if at least 30% of the markers showed instability and MSI-L/MSS if less than 30% of the makers showed instability. MSI status could be determined for 3639 CRC cases (93.4%).

Most studies used Methylight (42) methylation analysis to determine CIMP status, classified as positive or negative based on either an 8- (CPS-II, EPIC, NHS, NSHDS) (43, 44) or 5-gene (CCFR, MCCS) (45–47) panel. The percent of methylated reference value was calculated to determine whether each gene was positive for methylation (generally percent of methylated reference  > 10). DACHS used a different 5-gene panel (48, 49) to determine CIMP status and based methylation on the presence or absence of the methylation-specific PCR product. DALS (50) determined CIMP status using a classic panel of CpG islands (51, 52). Specific genes included in each panel, details of calling methylation status, and number of methylated genes present to classify a tumor as CIMP-positive are outlined in Supplementary Table 2 (available online). CIMP status could be determined for 3453 CRC cases (88.6%).

Studies used PCR, sequencing, and immunohistochemistry techniques to assess *BRAF* and *KRAS* mutations, as detailed in the Supplementary Methods (available online). The majority of studies evaluated *BRAF* via V600E mutations in exon 15 and *KRAS* via mutations in codons 12 and 13, although a few evaluated additional loci. *BRAF* and *KRAS* status could be determined for 3564 (91.4%) and 3435 (88.1%) CRC cases, respectively.

Tumor pathways were defined as follows, consistent with previously suggested classifications (3, 8): 1) Adenoma-carcinoma (traditional) pathway (MSS/MSI-L, CIMP-negative, *BRAF* wild-type, *KRAS* wild-type), 2) alternate pathway (MSS/MSI-L, CIMP-negative, *BRAF* wild-type, *KRAS*-mutated), and 3) serrated pathway (CIMP-positive, *BRAF*-mutated, *KRAS* wild-type). Tumor pathway could be classified for 2401 CRC cases (61.6%).

Tumor location was obtained from registry and pathology reports. Location was grouped based on the International Classification of Diseases (ICD-9) codes as follows: 1) Proximal (153.0/Hepatic flexure, 153.1/Transverse colon, 153.4/Cecum, 153.6/Ascending colon), 2) distal (153.2/Descending colon, 152.3/Sigmoid colon, 153.7/Splenic flexure), and 3) rectal (154.0/Rectosigmoid junction, 154.1/Rectum). Tumor location could be classified for 3808 CRC cases (97.7%).

### Statistical Analysis

**Figure 1.**
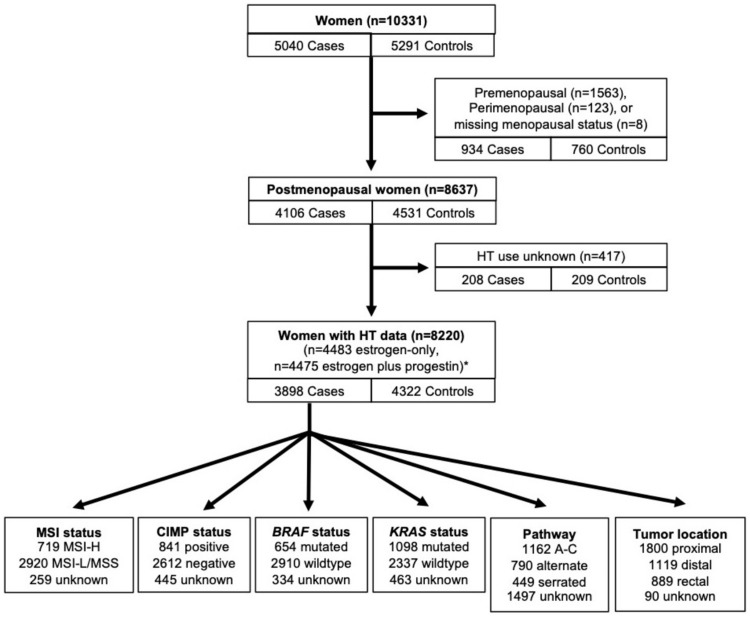
Overview of participants included in analytic population. A-C = adenoma-carcinoma; CIMP = CpG island methylator phenotype; HT = postmenopausal hormone therapy; MSI = microsatellite instability. *Estrogen-only and estrogen plus progestin groups are not mutually exclusive.

We excluded women who were pre- or perimenopausal at study baseline (934 cases, 760 controls) and those with missing data on HT use (208 cases, 209 controls). After exclusions, 3898 CRC cases and 4322 controls were included in our analyses (Figure 1).

Odds ratios (ORs) and 95% confidence intervals (CIs) from logistic regression models were used to approximate the relative risks for the association of HT use and CRC. Separate models were evaluated for each tumor-specific outcome using multinomial logistic regression with tumor marker status vs control as the outcome (eg, *BRAF*-mutated or *BRAF* wild-type vs control). All models included study site as well as covariates selected a priori based on known associations with both HT and CRC. These included age in years, body mass index (BMI; normal or underweight [BMI <25], overweight [BMI 25–30], obese [BMI >30], unknown), smoking status (current, former, never, unknown), and first-degree relative with CRC (yes, no, unknown). Secondary analyses were conducted for estrogen-only therapy and combined estrogen plus progestin therapy. For multinomial logistic regression models, Wald χ^2^ tests were used to evaluate heterogeneity in odds ratios by tumor marker status (53).

Additionally, sensitivity analyses were conducted excluding 1) women aged 45 years and younger (n = 131) and 2) women with probable Lynch syndrome based on 4 tumor markers (defined as MSI-H, CIMP-negative, *BRAF* wild-type, *KRAS* wild-type; n = 89), because both populations may have unique factors altering their CRC risk. We also performed a meta-analysis of the association of any HT use and CRC risk to evaluate heterogeneity by study site.

All analyses were conducted using R version 3.5.2 with a 2-sided *P* less than .05 considered statistically significant.

## Results

Baseline population characteristics of the 8220 postmenopausal women in our study are shown in Table 1. Compared with controls, cases were more likely to have a family history of CRC and be current or former smokers. Cases were less likely to be HT users than controls (32.4% vs 42.8%). Among those with formulation-specific data, cases were less likely than controls to use both estrogen-only (22.2% vs 29.7%) and estrogen plus progestin formulations (14.3% vs 17.8%).

**Table 1.** Baseline characteristics of 8220 postmenopausal women by case-control status

Characteristics^a^	Overall	Case	Control
	(n = 8220)	(n = 3898)	(n = 4322)
Age, mean (SD), y	65.28 (9.08)	64.79 (9.54)	65.72 (8.62)
Age group, y			
<45	101 (1.2)	84 (2.2)	17 (0.4)
45–55	828 (10.1)	464 (11.9)	364 (8.4)
55–65	2780 (33.8)	1246 (32.0)	1534 (35.5)
65–75	3309 (40.3)	1561 (40.0)	1748 (40.4)
>75	1202 (14.6)	543 (13.9)	659 (15.2)
First-degree relative with CRC			
Yes	1251 (15.2)	722 (18.5)	529 (12.2)
No	6633 (80.7)	2994 (76.8)	3639 (84.2)
Missing	336 (4.1)	182 (4.7)	154 (3.6)
Body mass index			
Normal or underweight	3659 (44.5)	1613 ( 41.4)	2046 (47.3)
Overweight	2818 (34.3)	1322 (33.9)	1496 (34.6)
Obese	1571 (19.1)	870 (22.3)	701 (16.2)
Missing	172 ( 2.1)	93 (2.4)	79 (1.8)
Smoking			
Current smoker	948 (11.5)	522 (13.4)	426 (9.9)
Former smoker	2619 (31.9)	1285 (33.0)	1334 (30.9)
Never smoker	4477 (54.5)	2012 (51.6)	2465 (57.0)
Missing	176 (2.1)	79 (2.0)	97 (2.2)
Self-reported race			
White	8077 (98.3)	3780 (97.0)	4297 (99.4)
Other	113 (1.4)	98 (2.5)	15 (0.4)
Missing	30 (0.4)	20 (0.5)	10 (0.2)
Study			
CCFR	1985 (24.1)	1215 ( 31.2)	770 (17.8)
CPSII	893 (10.9)	412 ( 10.6)	481 (11.1)
DACHS	2074 (25.2)	872 ( 22.4)	1202 (27.8)
DALS	891 (10.8)	427 (11.0)	464 (10.7)
EPIC Sweden	129 (1.6)	37 (0.9)	92 (2.1)
MCCS	455 (5.5)	185 (4.7)	270 (6.2)
NHS	1649 (20.1)	686 (17.6)	963 (22.3)
NSHDS	144 (1.8)	64 (1.6)	80 (1.9)
Any postmenopausal hormone therapy use			
Ever	3112 (37.9)	1262 (32.4)	1850 (42.8)
Never	5108 (62.1)	2636 (67.6)	2472 (57.2)
Estrogen-only			
Ever	1160 (14.1)	506 (13.0)	654 (15.1)
Never	3323 (40.4)	1778 (45.6)	1545 (35.7)
Missing	3737 (45.5)	1614 (41.4)	2123 (49.1)
Estrogen plus progestin			
Ever	717 (8.7)	328 (8.4)	389 (9.0)
Never	3758 (45.7)	1961 (50.3)	1797 (41.6)
Missing	3745 (45.6)	1609 (41.3)	2136 (49.4)

a No. (%) shown unless otherwise indicated. CCFR = Colon Cancer Family Registry; CPSII = Cancer Prevention Study II; CRC = colorectal cancer; DACHS = Darmkrebs: Chancen der Verhutung durch Screening Study; DALS = Diet Activity and Lifestyle Study; EPIC = European Prospective Investigation into Cancer; MCCS = Melbourne Collaborative Cohort Study; NHS = Nurses’ Health Study; NSHDS = Northern Sweden Health and Disease Study.

**Figure 2.**
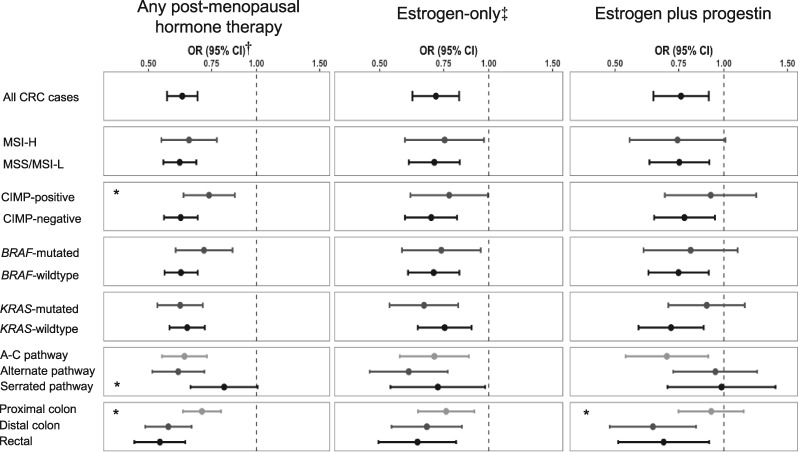
Association between postmenopausal hormone therapy (HT) use and colorectal cancer (CRC), overall and formulation specific. A-C = adenoma-carcinoma; CI = confidence interval; CIMP = CpG island methylator phenotype; CRC = colorectal cancer; MSI = microsatellite instability; OR = odds ratio. *Wald *P* < 0.05. Wald *P* values are comparing within-group odds ratios; reference groups are *BRAF* wild-type, *KRAS* wild-type, CIMP negative, traditional, distal colon. ^†^Controls are used as reference for all odds ratios. All odds ratios are adjusted for age, body mass index, smoking status, and first-degree family history of CRC. ‡Formulation-specific data were only available for a subset of women (n = 4483 for estrogen-only; n = 4475 for estrogen plus progestin).

Multivariable-adjusted associations between HT use, HT formulation, and overall- and tumor marker–specific CRC risk are presented in Figure 2 and Supplementary Table 3 (available online). Ever use of HT was associated with a 38% reduction in CRC risk (OR = 0.62, 95% CI = 0.56 to 0.69). Both use of estrogen-only (OR = 0.71, 95% CI = 0.62 to 0.83) and estrogen plus progestin (OR = 0.76, 95% CI = 0.64 to 0.91) formulations were associated with reduced CRC risk, although the effect estimates were attenuated compared with any HT use in this subsample of the study population. Few differences in baseline characteristics were noted between women with and without formulation-specific data (Supplementary Table 4, available online).

Among cases with respective tumor marker data, 19.8% were MSI-H (n = 719), 24.3% were CIMP-positive (n = 841), 18.4% were *BRAF*-mutated (n = 654), and 32.0% were *KRAS*-mutated (n = 1098). Ever use of HT was associated with reduced risk of almost all tumor marker subtypes of CRC, with some variation across subtypes (Figure 2; Supplementary Table 3, available online). The association of ever HT use and CRC was attenuated among CIMP-positive cases (OR = 0.74, 95% CI = 0.63 to 0.87) compared with CIMP-negative cases (OR = 0.62, 95% CI = 0.55 to 0.69) (*P*_het_ = .04). This trend was consistent across HT formulations, although the difference in odds ratios was not statistically significant. HT use was inversely associated with both *KRAS*-mutated and wild-type individuals. This association was consistent for estrogen-only use; however, estrogen plus progestin formulations were not statistically significantly associated with *KRAS*-mutated individuals (OR = 0.90, 95% CI = 0.70 to 1.14; *P*_het_ = 0.09). No differences were observed for MSI or *BRAF* mutation status.

Of 2401 tumors (61.6%) that were able to be classified by pathway, the majority were classified as adenoma-carcinoma pathway tumors (48.4%; n = 1162), with 32.9% classified as alternate pathway (n = 790) and 18.7% as the serrated pathway (n = 449). No major differences in baseline characteristics were noted between women who were and were not able to be classified by pathway (Supplementary Table 4, available online). The effect estimates for HT use in both the adenoma-carcinoma and alternate pathways were similar to that seen for HT overall (adenoma-carcinoma OR = 0.63, 95% CI = 0.55 to 0.73; alternate OR = 0.61, 95% CI = 0.51 to 0.72). However, the effect estimate was attenuated and no longer statistically significant for tumors that arose via the serrated pathway (OR = 0.81, 95% CI = 0.66 to 1.01; *P*_het_ vs adenoma-carcinoma = .04). This difference was not consistent across HT formulation: for estrogen-only formulations, ever use was statistically significantly inversely associated with all 3 pathways (adenoma-carcinoma OR = 0.71, 95% CI = 0.57 to 0.88; alternate OR = 0.60, 95% CI = 0.47 to 0.77; serrated OR = 0.72, 95% CI = 0.54 to 0.98), and there was no statistical difference between pathways. However, for estrogen plus progestin formulations, ever use was only statistically significantly associated with tumors that arose via the adenoma-carcinoma pathway (OR = 0.70, 95% CI = 0.55 to 0.91).

Most tumors were located in the proximal colon (47.3%), with tumors of the distal colon (29.4%) only slightly more common than rectal tumors (23.3%). Compared with distal colon (OR = 0.57, 95% CI = 0.49 to 0.66) and rectal tumors (OR = 0.54, 95% CI = 0.46 to 0.63), the effect estimate for the association of HT use and proximal colon tumors was attenuated (OR = 0.71, 95% CI = 0.62 to 0.80; *P*_het_ vs distal = .01). This trend was consistent across HT formulations, although there was no statistical difference between proximal and distal colon tumors for estrogen-only formulation (*P*_het_ =.32).

No substantial changes in results were noted after removing either the 131 women aged 45 years and younger or the 89 womenwith molecularly defined Lynch syndrome (Supplementary Tables 5 and 6, available online). Meta-analysis results were consistent with our pooled main analysis (summary OR = 0.64, 95% CI = 0.58 to 0.71; *P*_het _=.10).

## Discussion

In this large pooled study of postmenopausal women, HT use, regardless of formulation type, was associated with a decreased risk of CRC, consistent with prior research (11–20). In general, this inverse association was observed irrespective of MSI, CIMP, *BRAF*, or *KRAS* status. However, when considering all tumor markers together and grouping cases by common pathways and tumor location, the association was attenuated for tumors arising via the serrated pathway and for proximal colon tumors.

Our results do not support the hypothesis that the association of HT and CRC differs by the individual tumor markers MSI, *BRAF*, and *KRAS*. Strong inverse associations were observed for HT use and CRC, regardless of *BRAF* and *KRAS* status. Prior studies found a nearly 20% reduced risk among ever HT users irrespective of *BRAF* and *KRAS* mutation status, although effect estimates did not reach statistical significance (22, 23). These studies had substantially smaller samples sizes than ours, contributing to reduced power to detect difference in effect. We additionally observed strong inverse associations for HT use and both MSI-L/MSS and MSI-H CRC. Prior research is somewhat conflicting regarding the association of HT and MSI status, with most studies suggesting an association only among MSI-L/MSS patients (22, 24, 54). There are many possible explanations for this discrepancy, including sample size, study design, reference period used for ascertaining HT use, and panels used to classify MSI status. Because prior studies have indicated high concordance across MSI panels (41), we suspect the latter had the least influence.

We found some evidence that the association of HT differs by CIMP status, with an attenuated effect estimate observed for CIMP-positive tumors. A previous study had similar findings, reporting a borderline inverse association among CIMP-negative tumors and no association for CIMP-positive tumors (22, 47). This finding should be interpreted with caution because CIMP is not consistently defined across studies, and CIMP prevalence may be affected by detection method and sample quality.

Our results suggest that a comprehensive approach of considering tumor markers together as pathways may reveal otherwise nebulous patterns. Our findings indicate that the association of HT use and CRC was largely driven by tumors arising via the adenoma-carcinoma and alternate pathway. These tumors make up the majority of CRC cases, whereas serrated tumors represent about 20%–30% of CRC (1, 3, 8, 55–57). Serrated tumors, characterized as CIMP-positive, *BRAF*-mutated, and *KRAS* wild-type, tend to behave more aggressively, with faster progression and poorer prognosis (3, 8, 10, 56, 58–60). Based on the different biologic behavior, appearance, distribution of tumor markers, and genetic susceptibility of serrated tumors, it is plausible that HT may indeed play a lesser role in their pathogenesis.

We also observed a weaker association for tumors of the proximal colon, consistent with prior studies (22, 24). There is evidence that serrated tumors are more likely to develop in the proximal colon (8, 61, 62), so it is unclear whether these are independent associations. In our study, most serrated tumors (n = 391) were in the proximal colon. The association of HT use and proximal tumors was similar (OR = 0.68, 95% CI = 0.60 to 0.78) after removing serrated tumors from analysis, suggesting an independent association. However, 43.1% (n = 776) of proximal tumors could not be classified by pathway because of incomplete tumor marker data, so this analysis is limited. The proximal and distal colon have different embryologic origins, microbiomes, and microenvironments (62–66). As such, they appear to be predisposed to different tumor types. For instance, proximal colon tumors are more likely to be MSI-H, CIMP-positive, and mucinous and occur more commonly in women and older individuals (67–71). Further research is needed to better elucidate whether differences in the proximal colon make it less sensitive to the effects of estrogens (ie, fewer receptors, different microbiota), whether precancerous lesions in the proximal colon are estrogen insensitive based on differences in the carcinogenic pathway, or some combination of factors.

Our results indicate that both estrogen-only and estrogen plus progestin formulations reduce CRC risk. In general, effect estimates were attenuated for estrogen plus progestin formulations compared with estrogen-only formulations, perhaps reflecting smaller exposure frequencies. However, overall trends were similar. Two main exceptions were present. First, whereas estrogen-only and any HT use were associated with about a 40% reduction in tumors arising via the alternate pathway, estrogen plus progestin use was not statistically significantly associated with these tumors. This may indicate that alternate pathway tumorigenesis is specifically modified by estrogen and not progestin. Likewise, there was a null association between estrogen plus progestin use and proximal colon tumors despite a 24%–29% reduction in risk with estrogen-only or any HT, respectively.

To our knowledge, this is the largest study to assess whether the association of HT use and CRC differs by individual tumor markers and location. In addition, it is one of few investigations that combines multiple tumor markers to evaluate tumor pathway–specific associations. Some limitations should be considered in interpreting our results. First, all exposure and epidemiologic covariate information included in this analysis was based on self-report, which could lead to exposure misclassification. Second, HT use was assessed only during the reference period, and detailed information on dose, frequency, and duration of use was not routinely available. Third, we were not able to assess endogenous hormones that may reflect age at menarche, parity, or breast feeding, which may also influence CRC risk. Fourth, there is some evidence that HT users may be more likely to undergo CRC screening (72, 73). It is unclear how this may impact our results because this relationship may be complicated by differences in sensitivity of screening detection for specific CRC subtypes. Temporal trends and regional differences in screening and HT use may also influence observed associations. Finally, although this study includes populations in many locales, the participants were predominantly white, and therefore, these findings may not be generalizable to other racial and ethnic groups.

In this large, multisite study we observed a strong inverse association between HT use and CRC risk, regardless of individual tumor markers and HT formulation. The decreased risk may predominantly reflect tumors of the distal colon or rectum and those arising via the adenoma-carcinoma (traditional) pathway, because the association was relatively weaker among proximal colon tumors and those arising via the serrated pathway. Further investigation into the mechanisms underlying these differences may add to our understanding of subtype-specific CRC risk and pathways of tumorigenesis.

## Funding

Genetics and Epidemiology of Colorectal Cancer Consortium: This work was supported by the National Cancer Institute, National Institutes of Health, US Department of Health and Human Services R01 CA176272, U01 CA137088, and U01 CA164930. This research was funded in part through the National Institutes of Health/National Cancer Institute Cancer Center Support Grant P30 CA015704.

The Colon Cancer Family Registry (CCFR) was supported in part by National Cancer Institute/National Institutes of Health award number U01 CA167551 and through National Cancer Institute/National Institutes of Health U01/U24 cooperative agreements with the following CCFR sites: Ontario (OFCCR) (CA074783), Seattle (SCCFR) (CA074794 and R01 CA076366), and Australasian (ACCFR) (CA074778 and CA097735). The content of this manuscript does not necessarily reflect the views or policies of the National Cancer Institute or any of the collaborating centers in the CCFR, nor does mention of trade names, commercial products, or organizations imply endorsement by the US government, any cancer registry, or the CCFR.

CPS-II: The American Cancer Society funds the creation, maintenance, and updating of the Cancer Prevention Study II cohort. This study was conducted with Institutional Review Board approval.

DACHS: This work was supported by the German Research Council (BR 1704/6–1, BR 1704/6–3, BR 1704/6–4, CH 117/1–1, HO 5117/2–1, HE 5998/2–1, KL 2354/3–1, RO 2270/8–1, and BR 1704/17–1), the Interdisciplinary Research Program of the National Center for Tumor Diseases, Germany, and the German Federal Ministry of Education and Research (01KH0404, 01ER0814, 01ER0815, 01ER1505A, and 01ER1505B).

DALS: National Institutes of Health (R01 CA48998).

EPIC: The coordination of EPIC is financially supported by the European Commission (DGSANCO) and the International Agency for Research on Cancer. The national cohorts are supported by Danish Cancer Society (Denmark), Swedish Cancer Society, Swedish Research Council, and County Councils of Skåne and Västerbotten (Sweden).

MCCS: This cohort recruitment was funded by VicHealth and Cancer Council Victoria. The MCCS was further supported by Australian National Health and Medical Research Council grants 509348, 209057, 251553, and 504711 and by infrastructure provided by Cancer Council Victoria. Cases, and their vital status were ascertained through the Victorian Cancer Registry and the Australian Institute of Health and Welfare, including the National Death Index and the Australian Cancer Database.

Harvard cohort (NHS): NHS is supported by the National Institutes of Health (R01 CA137178, P01 CA087969, UM1 CA186107, R01 CA151993, R35 CA197735, K07 CA190673, and P50 CA127003).

NSHDS: Swedish Cancer Society; Cancer Research Foundation in Northern Sweden; Swedish Research Council; J C Kempe Memorial Fund; Faculty of Medicine, Umeå University, Umeå, Sweden; and Cutting-Edge Research Grant from the County Council of Västerbotten, Sweden.

Fred Hutchinson Cancer Research Center investigators were also supported by National Institutes of Health T32 CA094880 and National Institutes of Health K05 CA152715.

## Notes

**Role of the funders:** The funders had no role in the design of the study; the collection, analysis, and interpretation of the data; the writing of the manuscript; and the decision to submit the manuscript for publication.

**Authors contributions: **Conceptualization: JDL, MH, PAN; Data curation: TAH, BB, YL, UP; Formal analysis: JDL, BB, LH, YL, WS; Funding acquisition: HB, PTC, ATC, JC, JCF, SJG, GGG, MJG, MH, MAJ, RLM, VM, NM, SO, LCS, MLS, SNT, BV, UP, PAN; Investigation: all authors; Methodology: JDL, LH; Project administration: TAH, UP; Resources: HB, PTC, ATC, JC, JCF, SJG, GGG, MJG, MH, MAJ, RLM, VM, NM, SO, LCS, MLS, SNT, BV, UP, PAN; Supervision: UP, PAN; Visualization: JDL; Writing - original draft: JDL, TAH, UP, PAN; Writing - review & editing: all authors.

**Disclosures:** The authors have no conflicts of interest to disclose.

**Acknowledgments:** SCCFR: The authors would like to thank the study participants and staff of the Hormones and Colon Cancer and Seattle Cancer Family Registry studies (CORE studies). CPS-II: The authors thank the CPS-II participants and Study Management Group for their invaluable contributions to this research. The authors would also like to acknowledge the contribution to this study from central cancer registries supported through the Centers for Disease Control and Prevention National Program of Cancer Registries and cancer registries supported by the National Cancer Institute Surveillance Epidemiology and End Results program. DACHS: We thank all participants and cooperating clinicians, and Ute Handte-Daub, Utz Benscheid, Muhabbet Celik, and Ursula Eilber for excellent technical assistance. EPIC: Where authors are identified as personnel of the International Agency for Research on Cancer/World Health Organization, the authors alone are responsible for the views expressed in this article, and they do not necessarily represent the decisions, policy, or views of the International Agency for Research on Cancer/World Health Organization. Harvard cohort (NHS): The study protocol was approved by the institutional review boards of the Brigham and Women’s Hospital and Harvard T.H. Chan School of Public Health, and those of participating registries as required. We would like to thank the participants and staff of the NHS for their valuable contributions as well as the following state cancer registries for their help: AL, AZ, AR, CA, CO, CT, DE, FL, GA, ID, IL, IN, IA, KY, LA, ME, MD, MA, MI, NE, NH, NJ, NY, NC, ND, OH, OK, OR, PA, RI, SC, TN, TX, VA, WA, WY. The authors assume full responsibility for analyses and interpretation of these data. MCCS: Melbourne Collaborative Cohort Study cohort recruitment was funded by VicHealth and Cancer Council Victoria. The MCCS was further augmented by infrastructure provided by Cancer Council Victoria. Cases and their vital status were ascertained through the Victorian Cancer Registry and the Australian Institute of Health and Welfare, including the National Death Index and the Australian Cancer Database. NSHDS: The NSHDS investigators thank the Biobank Research Unit at Umeå University, the Västerbotten Intervention Programme, the Northern Sweden MONICA study, and Region Västerbotten for providing data and samples and acknowledge the contribution from Biobank Sweden, supported by the Swedish Research Council (VR 2017–00650).

## Supplementary Material

pkaa042_Supplementary_Data
