# Supplementary material for: Postmenopausal Hormone Therapy and Colorectal Cancer Risk by Molecularly Defined Subtypes and Tumor Location
Source: JNCI Cancer Spectr. 2020 May 19;4(5):pkaa042. doi: 10.1093/jncics/pkaa042 (PMC7477374; doi:10.1093/jncics/pkaa042)
Supplement: pkaa042_Supplementary_Data [file pkaa042_supplementary_data.pdf]

Supplementary Material

Table of Contents

SUPPLEMENTARY METHODS **2**

Description of Study Populations 2

Cancer Prevention Study II (CPS II)[1, 2] *2*

Colon Cancer Family Registry (CCFR)[3] *2*

Darmkrebs: Chancen der Verhütung durch Screening (DACHS)[4, 5] *3*

Diet, Activity, and Lifestyle Study (DALS)[6] *4*

European Prospective Investigation into Cancer (EPIC) - Sweden[7] *4*

Melbourne Collaborative Cohort Study (MCCS)[8, 9] *5*

Nurses' Health Study (NHS)[10] *5*

Northern Swedish Health and Disease Study (NSHDS)[11, 12] *6*

Additional Methods for Tumor Marker Detection 6

Microsatellite Instability (MSI) Status *6*

BRAF Somatic Mutation Status *7*

KRAS Somatic Mutation Status *8*

Harmonization of Environmental Data 8

SUPPLEMENTARY TABLES **10**

Supplementary Table 1. Study-specific markers used to assess microsatellite instability (MSI) and definition of MSI status 10

Supplementary Table 2. Study-specific panels used to assess CpG island methylator phenotype (CIMP) status 12

Supplementary Table 3. Case-control associations between post-menopausal hormone therapy (HT) at baseline and colorectal cancer (CRC), overall and by molecular subtype 13

Supplemental Table 4. Baseline characteristics of 1) 8,637 post-menopausal women, comparing women in our analytical population to women who were excluded due to missing postmenopausal hormone therapy (HT) data; 2) 8,220 post-menopausal women with HT data, comparing women with formulation-specific HT data to those without; 3) 3,898 post-menopausal CRC cases, comparing women who could be classified by pathway to those who could not 15

Supplementary Table 5. Sensitivity analysis for associations between post-menopausal hormone therapy (HT) at baseline and colorectal cancer (CRC), overall and by molecular subtype, removing 131 individuals < 45 years of age 18

Supplementary Table 6. Sensitivity analysis for associations between post-menopausal hormone therapy (HT) at baseline and colorectal cancer (CRC), overall and by molecular subtype, removing 89 individuals with molecularly defined Lynch Syndrome 20

REFERENCES **22**

Supplementary Methods

Description of Study Populations

The Genetics and Epidemiology of Colorectal Cancer Consortium (GECCO) is a collaborative effort comprised of a coordinating center and scientific researchers from well-characterized cohort and case-control studies conducted in North America and Europe. Summary descriptions and study participant inclusions/exclusion criteria for each of the studies included in our analysis are detailed below. For all studies, only participants with available tumor molecular characterization were included in analysis.

Cancer Prevention Study II (CPS II)[1, 2]

The CPS II Nutrition cohort is a prospective study of cancer incidence and mortality in the United States, established in 1992. At enrollment, participants completed a mailed self-administered questionnaire including information on demographic, medical, diet, and lifestyle factors. Follow-up questionnaires to update exposure information and to ascertain newly diagnosed cancers were sent biennially starting in 1997. Reported cancers were verified through medical records, state cancer registry linkage, or death certificates. Controls were matched on race, gender, and age. The Emory University Institutional Review Board approves all aspects of the CPS II Nutrition Cohort.

Colon Cancer Family Registry (CCFR)[3]

The CCFR (www.coloncfr.org) is a National Cancer Institute-supported consortium consisting of six centers dedicated to the establishment of a comprehensive collaborative infrastructure for interdisciplinary studies in the genetic epidemiology of colorectal cancer. The

CCFR includes data from approximately 42,500 total subjects (10,500 case probands and 26,900 unaffected and affected relatives, 4,280 unrelated population-based controls, and 920 spouse). Cases and unaffected controls, age 20 to 74 years, were recruited at the six participating centers beginning in 1998. All participants self-completed a standardized questionnaire that asked about established and suspected risk factors for colorectal cancer, including questions on medical history and medication use, reproductive history (for female participants), family history, physical activity, demographics, alcohol and tobacco use, and dietary factors. Participants from three participating centers (Seattle, Australia, Ontario) were included in this study.

Darmkrebs: Chancen der Verhütung durch Screening (DACHS)[4, 5]

This German study was initiated as a large population-based case-control study in 2003 in the Rhine-Neckar-Odenwald region (southwest region of Germany) to assess the potential of endoscopic screening for reduction of CRC risk and to investigate etiologic determinants of disease, particularly lifestyle/environmental factors and genetic factors. Briefly, cases with a first diagnosis of invasive CRC (International Classification of Diseases 10 codes C18-C20) who were at least 30 years of age, German speaking, resident in the study region, and mentally and physically able to participate in a one-hour interview, were recruited by their treating physicians either in the hospital a few days after surgery, or by mail after discharge from the hospital. Cases were confirmed based on histologic reports and hospital discharge letters following diagnosis of CRC. All hospitals treating CRC cancer patients in the study region participated. Community-based controls were randomly selected from population registries, employing frequency matching with respect to age (5-year groups), sex, and county of residence. Controls without a history of CRC were contacted by mail and follow-up calls. During an in-person interview, data

were collected on demographics, medical history, family history of CRC, and various lifestyle factors, as were blood and mouthwash samples.

Diet, Activity, and Lifestyle Study (DALS)[6]

DALS was a population-based, case–control study of colon cancer. Participants were recruited between 1991 and 1994 from 3 locations: the Kaiser Permanente Medical Care Program of Northern California, an 8-county area in Utah, and the metropolitan Twin Cities area of Minnesota. Eligibility criteria for cases included age at diagnosis between 30 and 79 years, diagnosis with first primary colon cancer (International Classification of Disease for Oncology, Second Edition, 18.0 and 18.2–18.9) between October 1, 1991, and September 30, 1994, English speaking, and competency to complete the interview. Individuals with cancer of the rectosigmoid junction or rectum were excluded, as were those with a pathology report noting familial adenomatous polyposis, Crohn's disease, or ulcerative colitis. A rapid-reporting system was used to identify all incident cases of colon cancer, resulting in the majority of cases being interviewed within 4 months of diagnosis. Controls from the Kaiser Permanente Medical Care Program were selected randomly from membership lists. In Utah, controls younger than 65 years of age were selected randomly through random-digit dialing and driver license lists. Controls 65 years of age and older were selected randomly from Health Care Financing Administration lists. In Minnesota, controls were identified from Minnesota driver license or state identification lists.

European Prospective Investigation into Cancer (EPIC) - Sweden[7]

EPIC is an on-going multicenter prospective cohort study designed to investigate the associations between diet, lifestyle, genetic and environmental factors and various types of

cancer. Briefly, 521,448 participants (~70% women) mostly aged 35 years or above were recruited between 1992 and 2000. Participants were recruited from 23 study centers in ten European countries. The current study included participants from Sweden. All study participants provided written informed consent, and ethical approval for the EPIC study was obtained from the review boards of IARC and local participating centers. Cases were identified by linkage with the essentially complete Cancer Registry of Northern Sweden and were verified by a gastrointestinal pathologist. Controls were selected from the full cohort of individuals who were alive and free of cancer (except non-melanoma skin cancer) at the time of case diagnosis.

Melbourne Collaborative Cohort Study (MCCS)[8, 9]

The MCCS is a prospective study that recruited 41,514 healthy adult participants aged between 27 and 76 years (99% aged 40-69) from the Melbourne metropolitan area between 1990 and 1994. The goal of this study was to examine the role of lifestyle factors in the risk of cancer and heart disease. Incident cases of CRC were identified through linkage to population-based cancer registries in Australia. Cases include participants with a histopathological diagnosis of invasive colorectal adenocarcinoma following the baseline study visit, informed consent, and sufficient FFPE material for somatic testing. Study protocols were approved by the Human Research Ethics Committee at the Cancer Council Victoria.

Nurses' Health Study (NHS)[10]

The NHS cohort began in 1976 when 121,700 married female registered nurses age 30–55 years returned the initial questionnaire that ascertained a variety of important health-related exposures.1 Since 1976, follow-up questionnaires have been mailed every 2 years. Colorectal

cancer and other outcomes were reported by participants or next-of-kin and followed up through review of the medical and pathology record by physicians. Overall, more than 97% of self-reported colorectal cancers were confirmed by medical-record review. Information was abstracted on histology and primary location. The rate of follow-up evaluation has been high: as a proportion of the total possible follow-up time, follow-up evaluation has been more than 92%. Colorectal cancer cases were ascertained through June 1, 2008.

Northern Swedish Health and Disease Study (NSHDS)[11, 12]

NSHDS, which has been described in more detail elsewhere, comprises over 110,000 participants, including approximately one third with repeated sampling occasions, from three population-based cohorts: the Västerbotten Intervention Project (VIP), the Northern Sweden WHO Monitoring of Trends and Cardiovascular Disease (MONICA) Study, and the local Mammography Screening Project (MSP). The largest is the ongoing Västerbotten Intervention Programme, in which all residents of Västerbotten County were invited to a health examination upon turning 30 (some years), 40, 50 and 60 years of age. Extensive measured and self-reported health and lifestyle data were collected at the health exam.

Additional Methods for Tumor Marker Detection

Microsatellite Instability (MSI) Status

In addition to polymerase chain reaction as outlined in the manuscript and Supplemental Table 1, MSI testing was conducted using standardized immunohistochemical (IHC) detection of deficiency for mismatch repair gene proteins MLH1, MSH2, MSH6, and PMS2 for NSHDS and

EPIC[11] as well as a subset of CCFR[13, 14] and MCCS samples without PCR-based MSI characterization. MSI status was also determined using a mononucleotide marker panel[15] (DACHS) and a panel of two mononucleotides (BAT26 and TGFβRII) and 10 tetranucleotide repeats[16-18] (DALS). Both the methods used in DACHS and DALS have been shown to have high concordance with the Bethesda Consensus Panel.[19] Tumoral and normal DNA were PCR amplified with these 12 primer sets, and MSI was defined as $\geq$ 1 new PCR product either smaller or larger than those produced from normal DNA. For BAT26, the PCR product from tumor had to be $\geq$4 base pairs smaller than that from germline. For the tetranucleotide repeat panel, MSI status was based on $\geq$30% markers showing instability and MSI-L/MSS if <30% of repeats were unstable.

BRAF Somatic Mutation Status

BRAF V600E mutations were assessed using a fluorescent allele-specific PCR assay (CCFR and MCCS), PCR (CPS-II), real-time PCR using an allelic discrimination assay (EPIC and NSHDS),[20] PCR and pyrosequencing (NHS),[21, 22] and IHC analysis of V600E expression (DACHS). *BRAF* mutations were also assessed using amplification and sequencing of exon 15 on *BRAF* (DACHS and DALS). DACHS amplified exon 15 using FideliTaq polymerase and sequenced using the BigDye Terminator v1.1 Cycle Sequencing Kit on an ABI 3500 Genetic Analyzer.[5] DALS amplified exon 15 using Applied Biosystems AmpliTaq Gold and sequencing.[23]

KRAS Somatic Mutation Status

Mutations in *KRAS* codons 12 and 13 were assessed using Sanger sequencing (CCFR),[24, 25] PCR (CPS-II, also evaluated codon 14), BigDye v.3.1 sequencing (EPIC and NSHDS),[12] or a combination of methods. DACHS used either a single stranded conformational polymorphism technique or Sanger sequencing, as reported previously.[5] DALS evaluated *KRAS* mutations by amplifying codons 12 and 13 using Taq FS DNA polymerase and sequencing using prism BigDye terminators and cycle sequencing on an ABI prism 377 automated sequencer.[26] MCCS used real-time PCR with high resolution melting analysis followed by direct Sanger sequencing for positive cases.[27] NHS used real-time PCR and pyrosequencing of codons 12, 13, 61, and 146.[28, 29]

Harmonization of Environmental Data

We carried out a multi-step data harmonization procedure, reconciling each study's unique protocols and data-collection instruments at the GECCO coordinating center (Fred Hutchinson Cancer Research Center). First, we defined common data elements (CDEs). We examined the questionnaires and data dictionaries for each study to identify study-specific data elements that could be mapped to the CDEs. Through an iterative process, we communicated with each data contributor to obtain relevant data and coding information. The data elements were written to a common data platform, transformed via a SQL programming script, and combined into a single dataset with common definitions, standardized permissible values, and standardized coding. The mapping and resulting data were reviewed for quality assurance, and range and logic checks were performed to assess data distributions within and between studies.

Outlying samples were truncated to the minimum or maximum value of established range for each variable.

For CCFR, DALS, NHS, and NSHDS, post-menopausal hormone therapy use was ascertained as use at the reference period. The reference period time was defined as 2 years preceding enrollment for CCFR and DALS, use in 1990 for NHS, and current use for NSHDS. For CPS-II, DACHS, EPIC, and MCCS, post-menopausal hormone therapy use was ascertained as ever vs. never use.

Supplementary Tables

Supplementary Table 1. Study-specific markers used to assess microsatellite instability (MSI) and definition of MSI status

Study	Mononucleotide Markers	Dinucleotide Markers	Other Markers	Threshold for Interpretability	Definitions
CCFR	BAT25, BAT26, BAT40, BAT34C4	D5S346, D17S250, ACTC, D18S55, D10S197	MYCL	≥4 interpretable markers	* MSI-H if ≥30% markers showed instability<br>* MSI-L/MSS if <30% showed instability
CPS-II	BAT25, BAT26, BAT40, BAT34C4	ACTC, D10S197, D17S250, D18S55, D5S346	MYCL	≥5 interpretable markers (unless 4 markers were unstable)	* MSI-H if ≥30% markers showed instability<br>* MSI-L/MSS if <30% showed instability
DACHS	BAT25, BAT26, CAT25	N/A	N/A	All 3 markers interpretable	* MSI-H if ≥1 marker showed instability<br>* MSS if 0 markers showed instability
DALS	BAT26, TGFBRII	N/A	UT2127, UT2271, UT5144, UT868, UT5013, UT909, UT1205, UT5658, UT269, UT574	≥6 of 10 markers be interpretable from tetranucleotide repeat panel	* MSI: Instability in BAT26, TGFBRII, or 10 tetranucleotide marker panel.<br>- 10 marker panel: ≥30% unstable repeats.<br>* MSS: Stability in BAT26, TGFBRII, and 10-marker panel.<br>- 10 marker panel: <30% repeats were unstable.
MCCS	BAT25, BAT26, BAT40, BAT34C4	D5S346, D17S250, ACTC, D18S55, D10S197	MYCL	≥4 interpretable markers	* MSI-H if ≥30% markers showed instability<br>* MSI-L/MSS if <30% showed instability
NHS	BAT25, BAT26, BAT40	D18S55, D18S56, D18S67, D18S487, D2S123, D5S346, D17S250	-	>7 interpretable markers	* MSI-H if ≥30% markers showed instability<br>* MSI-L/MSS if <30% showed instability

EPIC and NSHDS utilized immunohistochemical detection of deficiency for mismatch repair gene proteins MLH1, MSH2, MSH6, and PMHS2

Abbreviations: CCFR, Colon Cancer Family Registry; CPSII, Cancer Prevention Study-II; DACHS, Darmkrebs: Chancen der Verhutung durch Screening Study; DALS - Diet Activity and Lifestyle Study; EPIC, European Prospective Investigation into Cancer; MCCS, Melbourne Collaborative Cohort Study; NHS, Nurses' Health Study; NSHDS, Northern Sweden Health and Disease Study

Supplementary Table 2. Study-specific panels used to assess CpG island methylator phenotype (CIMP) status

Study	Panel Genes	Marker Positive Definition	CIMP-positive	CIMP-negative
CCFR	CACNA1G, IGF2, NEUROG1, RUNX3, and SOCS1	PMR > 10	≥3 methylated markers	≤2 methylated markers
CPS-II	CDKN2A, MLH1, CACNA1G, NEUROG1, RUNX3, SOCS1, IGF2, CRABP1	PMR > 10	≥1 methylated markers	No methylated markers
DACHS	MGMT, MLH1, MINT1, MINT2, MINT31		≥1 methylated markers	No methylated markers
DALS	MINT1, MINT2, MINT31, CDKN2A9, and hMLH1		≥2 methylated markers	≤1 methylated markers
EPIC - Sweden	CDKN2A, MLH1, CACNA1G, NEUROG1, RUNX3, SOCS1, IGF2, CRABP1	PMR > 10	≥1 methylated markers	No methylated markers
MCCS	CACNA1G, IGF2, NEUROG1, RUNX3, and SOCS1	PMR > 10	≥3 methylated markers	≤2 methylated markers
NHS	CDKN2A, MLH1, CACNA1G, NEUROG1, RUNX3, SOCS1, IGF2, CRABP1	PMR > 4 for CDKN2A, MLH1, CACNA1G, NEUROG1, RUNX3, SOCS1. PMR > 6 for CRABP1, IGF2	≥1 methylated markers	No methylated markers
NSHDS	CDKN2A, MLH1, CACNA1G, NEUROG1, RUNX3, SOCS1, IGF2, CRABP1	PMR > 10	≥1 methylated markers	No methylated markers

Abbreviations: CCFR, Colon Cancer Family Registry; CPSII, Cancer Prevention Study-II; DACHS, Darmkrebs: Chancen der Verhutung durch Screening Study; DALS - Diet Activity and Lifestyle Study; EPIC, European Prospective Investigation into Cancer; MCCS, Melbourne Collaborative Cohort Study; NHS, Nurses' Health Study; NSHDS, Northern Sweden Health and Disease Study; PMR, percent of methylated reference

Supplementary Table 3. Case-control associations between post-menopausal hormone therapy (HT) at baseline and colorectal cancer (CRC), overall and by molecular subtype

	Any HT use (n=8,220)					Estrogen only (n=4,483)					Estrogen plus Progestin (n=4,475)				
	Ever user n	(%)	OR	(95% CI)	Wald p-value	Ever user n	(%)	OR	(95% CI)	Wald p-value	Ever user n	(%)	OR	(95% CI)	Wald p-value
Case	1262	(32)	0.62	(0.56 - 0.69)		506	(22)	0.71	(0.62 - 0.83)		328	(14)	0.76	(0.64 - 0.91)	
Control	1850	(43)				654	(30)				389	(18)			
MSI-H	230	(32)	0.65	(0.54 - 0.78)	0.516	103	(23)	0.76	(0.59 - 0.97)	0.613	62	(14)	0.74	(0.55 - 1.01)	0.941
MSS/MSI-L	944	(32)	0.61	(0.55 - 0.68)		368	(22)	0.71	(0.60 - 0.83)		242	(14)	0.75	(0.62 - 0.91)	
CIMP-positive	284	(34)	0.74	(0.63 - 0.87)	0.038	107	(24)	0.78	(0.61 - 1.00)	0.377	71	(16)	0.92	(0.69 - 1.23)	0.274
CIMP-negative	860	(33)	0.62	(0.55 - 0.69)		329	(22)	0.69	(0.59 - 0.82)		226	(15)	0.78	(0.64 - 0.94)	
BRAF -mutated	223	(34)	0.71	(0.60 - 0.86)	0.124	101	(23)	0.74	(0.58 - 0.95)	0.708	64	(15)	0.81	(0.60 - 1.09)	0.621
BRAF -wildtype	950	(33)	0.62	(0.55 - 0.69)		367	(22)	0.70	(0.60 - 0.83)		242	(14)	0.75	(0.62 - 0.91)	
KRAS -mutated	361	(33)	0.61	(0.53 - 0.71)	0.570	138	(21)	0.66	(0.53 - 0.82)	0.258	110	(17)	0.90	(0.70 - 1.14)	0.088
KRAS -wildtype	773	(33)	0.64	(0.57 - 0.72)		310	(24)	0.76	(0.64 - 0.90)		181	(14)	0.71	(0.58 - 0.88)	
A-C pathway	392	(34)	0.63	(0.55 - 0.73)		145	(23)	0.71	(0.57 - 0.88)		90	(14)	0.70	(0.53 - 0.91)	
Alternate pathway	261	(33)	0.61	(0.51 - 0.72)	0.686	99	(20)	0.60	(0.47 - 0.77)	0.273	88	(18)	0.96	(0.73 - 1.25)	0.062
Serrated pathway	162	(36)	0.81	(0.66 - 1.01)	0.037	67	(23)	0.72	(0.54 - 0.98)	0.897	49	(17)	1.00	(0.71 - 1.41)	0.079
Proximal colon	626	(35)	0.71	(0.62 - 0.80)	0.010	254	(24)	0.76	(0.64 - 0.91)	0.318	180	(17)	0.92	(0.75 - 1.14)	0.013
Distal colon	340	(30)	0.57	(0.49 - 0.66)		132	(21)	0.67	(0.54 - 0.84)		77	(12)	0.64	(0.48 - 0.84)	
Rectal	271	(30)	0.54	(0.46 - 0.63)	0.585	103	(20)	0.64	(0.50 - 0.81)	0.687	69	(13)	0.68	(0.51 - 0.91)	0.704

Abbreviations: MSI, microsatellite instability; CIMP, CpG island methylator phenotype; A-C, adenoma-carcinoma

*All ORs use the control group as the reference

†Wald p-values are comparing within-group ORs; reference groups are: *BRAF*-wildtype, *KRAS*-wildtype, CIMP-negative, traditional pathway, distal colon

Supplemental Table 4. Baseline characteristics of 1) 8,637 post-menopausal women, comparing women in our analytical population to women who were excluded due to missing postmenopausal hormone therapy (HT) data; 2) 8,220 post-menopausal women with HT data, comparing women with formulation-specific HT data to those without; 3) 3,898 post-menopausal CRC cases, comparing women who could be classified by pathway to those who could not

	Women without HT (n=417)	Analytic Population (n=8220)	Missing E + P data (n=3745)	E+P data available (n=4475)	Missing E only data (n=3737)	E only data available (n=4483)	Missing pathway data (n=881)	Pathway data available (n=3017)
Age, mean (standard deviation)	62.43 (7.37)	65.28 (9.08)	67.89 (9.23)	63.09 (8.35)	67.87 (9.30)	63.12 (8.30)	62.32 (11.06)	65.50 (8.93)
Age group								
<45 years	3 (0.7)	101 (1.2)	13 (0.3)	88 (2.0)	15 (0.4)	86 (1.9)	53 (6.0)	31 (1.0)
45-55 years	30 (7.2)	828 (10.1)	235 (6.3)	593 (13.3)	241 (6.4)	587 (13.1)	169 (19.2)	295 (9.8)
55-65 years	254 (60.9)	2780 (33.8)	1113 (29.7)	1667 (37.3)	1102 (29.5)	1678 (37.4)	230 (26.1)	1016 (33.7)
65-75 years	100 (24.0)	3309 (40.3)	1472 (39.3)	1837 (41.1)	1466 (39.2)	1843 (41.1)	324 (36.8)	1237 (41.0)
>75 years	30 (7.2)	1202 (14.6)	912 (24.4)	290 (6.5)	913 (24.4)	289 (6.4)	105 (11.9)	438 (14.5)
First-degree relative with CRC								
Yes	31 (7.4)	1251 (15.2)	468 (12.5)	783 (17.5)	470 (12.6)	781 (17.4)	152 (17.3)	570 (18.9)
No	318 (76.3)	6633 (80.7)	3110 (83.0)	3523 (78.7)	3099 (82.9)	3534 (78.8)	684 (77.6)	2310 (76.6)
Missing	68 (16.3)	336 (4.1)	167 (4.5)	169 (3.8)	168 (4.5)	168 (3.7)	45 (5.1)	137 (4.5)
Body mass index								
Normal or underweight	157 (37.6)	3659 (44.5)	1603 (42.8)	2056 (45.9)	1598 (42.8)	2061 (46.0)	368 (41.8)	1245 (41.3)
Overweight	135 (32.4)	2818 (34.3)	1377 (36.8)	1441 (32.2)	1377 (36.8)	1441 (32.1)	294 (33.4)	1028 (34.1)
Obese	78 (18.7)	1571 (19.1)	714 (19.1)	857 (19.2)	713 (19.1)	858 (19.1)	188 (21.3)	682 (22.6)
Missing	47 (11.3)	172 (2.1)	51 (1.4)	121 (2.7)	49 (1.3)	123 (2.7)	31 (3.5)	62 (2.1)
Smoking								
Current smoker	43 (10.3)	948 (11.5)	376 (10.0)	572 (12.8)	372 (10.0)	576 (12.8)	133 (15.1)	389 (12.9)
Former smoker	107 (25.7)	2619 (31.9)	810 (21.6)	1809 (40.4)	817 (21.9)	1802 (40.2)	267 (30.3)	1018 (33.7)
Never smoker	191 (45.8)	4477 (54.5)	2398 (64.0)	2079 (46.5)	2387 (63.9)	2090 (46.6)	474 (53.8)	1538 (51.0)
Missing	76 (18.2)	176 (2.1)	161 (4.3)	15 (0.3)	161 (4.3)	15 (0.3)	7 (0.8)	72 (2.4)
Self-reported race								
White	413 (99.0)	8077 (98.3)	3742 (99.9)	4335 (96.9)	3732 (99.9)	4345 (96.9)	846 (96.0)	2934 (97.2)
Other	4 (1.0)	113 (1.4)	3 (0.1)	110 (2.5)	2 (0.1)	111 (2.5)	29 (3.3)	69 (2.3)
Missing	0 (0.0)	30 (0.4)	0 (0.0)	30 (0.7)	3 (0.1)	27 (0.6)	6 (0.7)	14 (0.5)
Study								
CCFR	62 (14.9)	1985 (24.1)	51 (1.4)	1934 (43.2)	41 (1.1)	1944 (43.4)	358 (40.6)	857 (28.4)
CPSII	18 (4.3)	893 (10.9)	1 (0.0)	892 (19.9)	3 (0.1)	890 (19.9)	159 (18.0)	253 (8.4)
DACHS	7 (1.7)	2074 (25.2)	2074 (55.4)	- -	2074 (55.5)	- -	170 (19.3)	702 (23.3)
DALS	14 (3.4)	891 (10.8)	891 (23.8)	- -	891 (23.8)	- -	111 (12.6)	316 (10.5)
EPIC Sweden	49 (11.8)	129 (1.6)	129 (3.4)	- -	129 (3.5)	- -	6 (0.7)	31 (1.0)
MCCS	1 (0.2)	455 (5.5)	455 (12.1)	- -	455 (12.2)	- -	20 (2.3)	165 (5.5)
NHS	71 (17.0)	1649 (20.1)	0 (0.0)	1649 (36.8)	0 (0.0)	1649 (36.8)	52 (5.9)	634 (21.0)
NSHDS	195 (46.8)	144 (1.8)	144 (3.8)	- -	144 (3.9)	- -	5 (0.6)	59 (2.0)
Any post-menopausal hormone therapy use								
Ever	- -	3112 (37.9)	1288 (34.4)	1824 (40.8)	1283 (34.3)	1829 (40.8)	239 (27.1)	1023 (33.9)
Never	- -	5108 (62.1)	2457 (65.6)	2651 (59.2)	2454 (65.7)	2654 (59.2)	642 (72.9)	1994 (66.1)
Case-control status								
Case	208 (49.9)	3898 (47.4)	1609 (43.0)	2289 (51.2)	1614 (43.2)	2284 (50.9)	881 (100.0)	3017 (100.0)
Control	209 (50.1)	4322 (52.6)	2136 (57.0)	2186 (48.8)	2123 (56.8)	2199 (49.1)	- -	- -

n (%) shown unless otherwise indicated

*Formulation-specific HT data were only available for CCFR, CPSII, and MCCS

†Abbreviations: CRC, colorectal cancer; CCFR, Colon Cancer Family Registry; CPSII, Cancer Prevention Study-II; DACHS, Darmkrebs: Chancen der Verhutung durch Screening Study; DALS - Diet Activity and Lifestyle Study; EPIC, European Prospective Investigation into Cancer; MCCS, Melbourne Collaborative Cohort Study; NHS, Nurses' Health Study; NSHDS, Northern Sweden Health and Disease Study

Supplementary Table 5. Sensitivity analysis for associations between post-menopausal hormone therapy (HT) at baseline and colorectal cancer (CRC), overall and by molecular subtype, removing 131 individuals < 45 years of age

	Any HT use (n=8,089)					Estrogen only (n=4,374)					Estrogen plus Progestin (n=4,362)				
	Ever user				Wald	Ever user				Wald	Ever user				Wald
	n	(%)	OR	(95% CI)	p-value	n	(%)	OR	(95% CI)	p-value	n	(%)	OR	(95% CI)	p-value
Case	1246	(33)	0.63	(0.57 - 0.69)		495	(23)	0.72	(0.62 - 0.84)		325	(15)	0.78	(0.65 - 0.93)	
Control	1841	(43)				650	(30)				388	(18)			
MSI-H	228	(32)	0.67	(0.56 - 0.80)	0.373	101	(23)	0.76	(0.59 - 0.98)	0.598	62	(14)	0.78	(0.57 - 1.06)	0.863
MSS/MSI-L	931	(33)	0.62	(0.55 - 0.69)		360	(23)	0.71	(0.61 - 0.84)		239	(15)	0.76	(0.63 - 0.92)	
CIMP-positive	282	(34)	0.74	(0.62 - 0.87)	0.047	107	(24)	0.78	(0.61 - 1.00)	0.364	70	(16)	0.91	(0.68 - 1.22)	0.310
CIMP-negative	856	(33)	0.62	(0.55 - 0.69)		327	(22)	0.70	(0.59 - 0.82)		225	(15)	0.78	(0.64 - 0.95)	
BRAF -mutated	222	(34)	0.73	(0.60 - 0.87)	0.108	101	(23)	0.76	(0.59 - 0.98)	0.588	63	(15)	0.82	(0.60 - 1.11)	0.666
BRAF -wildtype	935	(33)	0.62	(0.56 - 0.69)		356	(22)	0.71	(0.60 - 0.83)		240	(15)	0.76	(0.63 - 0.93)	
KRAS -mutated	358	(33)	0.62	(0.54 - 0.72)	0.612	136	(22)	0.67	(0.54 - 0.83)	0.275	110	(17)	0.91	(0.72 - 1.17)	0.078
KRAS -wildtype	761	(33)	0.65	(0.58 - 0.72)		302	(24)	0.76	(0.64 - 0.90)		178	(14)	0.72	(0.59 - 0.89)	
A-C pathway	390	(34)	0.63	(0.54 - 0.73)		145	(23)	0.72	(0.57 - 0.89)		89	(14)	0.69	(0.53 - 0.90)	
Alternate pathway	259	(33)	0.60	(0.51 - 0.71)	0.676	97	(20)	0.59	(0.46 - 0.76)	0.203	88	(18)	0.95	(0.73 - 1.24)	0.053
Serrated pathway	161	(36)	0.80	(0.65 - 1.00)	0.044	67	(23)	0.73	(0.54 - 0.98)	0.939	48	(17)	0.96	(0.68 - 1.36)	0.098
Proximal colon	618	(35)	0.71	(0.63 - 0.80)	0.006	249	(24)	0.77	(0.64 - 0.92)	0.268	179	(17)	0.94	(0.76 - 1.16)	0.008
Distal colon	335	(31)	0.56	(0.48 - 0.65)		128	(21)	0.67	(0.53 - 0.84)		76	(13)	0.63	(0.48 - 0.83)	
Rectal	268	(32)	0.56	(0.48 - 0.66)	0.997	101	(21)	0.67	(0.52 - 0.85)	0.999	68	(14)	0.71	(0.53 - 0.95)	0.529

Abbreviations: MSI, microsatellite instability; CIMP, CpG island methylator phenotype; A-C, adenoma-carcinoma

*All ORs use the control group as the reference

†Wald p-values are comparing within-group ORs; reference groups are: *BRAF*-wildtype, *KRAS*-wildtype, CIMP-negative, traditional pathway, distal colon

Supplementary Table 6. Sensitivity analysis for associations between post-menopausal hormone therapy (HT) at baseline and colorectal cancer (CRC), overall and by molecular subtype, removing 89 individuals with molecularly defined Lynch Syndrome

	Any HT use (n=8,131)					Estrogen only (n=4,431)					Estrogen plus Progestin (n=4,422)				
	Ever user				Wald	Ever user				Wald	Ever user				Wald
	n	(%)	OR	(95% CI)	p-value	n	(%)	OR	(95% CI)	p-value	n	(%)	OR	(95% CI)	p-value
Case	1230	(32)	0.62	(0.56 - 0.68)		491	(22)	0.71	(0.61 - 0.82)		323	(14)	0.77	(0.64 - 0.91)	
Control	1850	(43)				654	(30)				389	(18)			
MSI-H	198	(31)	0.63	(0.52 - 0.77)	0.712	88	(22)	0.72	(0.55 - 0.94)	0.887	57	(14)	0.78	(0.57 - 1.07)	0.794
MSS/MSI-L	944	(32)	0.61	(0.55 - 0.68)		368	(22)	0.71	(0.6 - 0.83)		242	(14)	0.75	(0.62 - 0.91)	
CIMP-positive	284	(34)	0.74	(0.63 - 0.87)	0.031	107	(24)	0.78	(0.61 - 1.00)	0.322	71	(16)	0.92	(0.68 - 1.23)	0.317
CIMP-negative	828	(33)	0.61	(0.55 - 0.68)		314	(21)	0.68	(0.58 - 0.81)		221	(15)	0.79	(0.65 - 0.96)	
BRAF -mutated	223	(34)	0.71	(0.60 - 0.86)	0.104	101	(23)	0.74	(0.58 - 0.95)	0.639	64	(15)	0.81	(0.60 - 1.09)	0.673
BRAF -wildtype	918	(33)	0.61	(0.55 - 0.68)		352	(22)	0.70	(0.59 - 0.82)		237	(14)	0.76	(0.62 - 0.92)	
KRAS -mutated	361	(33)	0.61	(0.53 - 0.71)	0.635	138	(21)	0.66	(0.53 - 0.83)	0.316	110	(17)	0.89	(0.70 - 1.14)	0.110
KRAS -wildtype	741	(33)	0.64	(0.57 - 0.71)		295	(23)	0.75	(0.63 - 0.89)		176	(14)	0.72	(0.58 - 0.89)	
A-C pathway	392	(34)	0.63	(0.55 - 0.73)		145	(23)	0.71	(0.57 - 0.88)		90	(14)	0.70	(0.53 - 0.91)	
Alternate pathway	261	(33)	0.61	(0.51 - 0.72)	0.686	99	(20)	0.60	(0.47 - 0.77)	0.273	88	(18)	0.96	(0.73 - 1.25)	0.062
Serrated pathway	162	(36)	0.81	(0.66 - 1.01)	0.037	67	(23)	0.72	(0.54 - 0.98)	0.897	49	(17)	1.00	(0.71 - 1.41)	0.079
Proximal colon	600	(35)	0.70	(0.62 - 0.79)	0.013	241	(23)	0.75	(0.62 - 0.90)	0.424	176	(17)	0.93	(0.76 - 1.15)	0.009
Distal colon	336	(30)	0.57	(0.49 - 0.66)		131	(21)	0.68	(0.54 - 0.85)		76	(12)	0.63	(0.48 - 0.83)	
Rectal	270	(31)	0.54	(0.46 - 0.63)	0.625	103	(20)	0.64	(0.50 - 0.82)	0.708	69	(13)	0.68	(0.51 - 0.91)	0.664

All ORs use the control group as the reference. Lynch Syndrome defined as MSI-H, CIMP-negative, *BRAF*-wildtype, *KRAS*-wildtype

Wald p-values are comparing within-group ORs; reference groups are: *BRAF*-wildtype, *KRAS*-wildtype, CIMP-negative, traditional pathway, distal colon

Abbreviations: MSI, microsatellite instability; CIMP, CpG island methylator phenotype; A-C, adenoma-carcinoma

References

1. Calle EE, Rodriguez C, Jacobs EJ, *et al.* The American Cancer Society Cancer Prevention Study II Nutrition Cohort: rationale, study design, and baseline characteristics. Cancer 2002;94(9):2490-501.

2. Campbell PT, Deka A, Briggs P, *et al.* Establishment of the cancer prevention study II nutrition cohort colorectal tissue repository. Cancer Epidemiol Biomarkers Prev 2014;23(12):2694-702.

3. Newcomb PA, Baron J, Cotterchio M, *et al.* Colon Cancer Family Registry: an international resource for studies of the genetic epidemiology of colon cancer. Cancer Epidemiol Biomarkers Prev 2007;16(11):2331-43.

4. Brenner H, Chang-Claude J, Jansen L, *et al.* Reduced risk of colorectal cancer up to 10 years after screening, surveillance, or diagnostic colonoscopy. Gastroenterology 2014;146(3):709-17.

5. Jia M, Jansen L, Walter V, *et al.* No association of CpG island methylator phenotype and colorectal cancer survival: population-based study. Br J Cancer 2016;115(11):1359-1366.

6. Slattery ML, Potter J, Caan B, *et al.* Energy balance and colon cancer--beyond physical activity. Cancer Res 1997;57(1):75-80.

7. Riboli E, Hunt KJ, Slimani N, *et al.* European Prospective Investigation into Cancer and Nutrition (EPIC): study populations and data collection. Public Health Nutr 2002;5(6B):1113-24.

8. Giles GG, English DR. The Melbourne Collaborative Cohort Study. IARC Sci Publ 2002;156:69-70.

9. Rosty C, Young JP, Walsh MD, *et al.* Colorectal carcinomas with KRAS mutation are associated with distinctive morphological and molecular features. Mod Pathol 2013;26(6):825-34.

10. Belanger CF, Hennekens CH, Rosner B, *et al.* The nurses' health study. Am J Nurs 1978;78(6):1039-40.

11. Dahlin AM, Palmqvist R, Henriksson ML, *et al.* The role of the CpG island methylator phenotype in colorectal cancer prognosis depends on microsatellite instability screening status. Clin Cancer Res 2010;16(6):1845-55.

12. Myte R, Gylling B, Haggstrom J, *et al.* One-carbon metabolism biomarkers and genetic variants in relation to colorectal cancer risk by KRAS and BRAF mutation status. PLoS One 2018;13(4):e0196233.

13. Lindor NM, Burgart LJ, Leontovich O, *et al.* Immunohistochemistry versus microsatellite instability testing in phenotyping colorectal tumors. J Clin Oncol 2002;20(4):1043-8.

14. Newcomb PA, Zheng Y, Chia VM, *et al.* Estrogen plus progestin use, microsatellite instability, and the risk of colorectal cancer in women. Cancer Res 2007;67(15):7534-9.

15. Findeisen P, Kloor M, Merx S, *et al.* T25 repeat in the 3' untranslated region of the CASP2 gene: a sensitive and specific marker for microsatellite instability in colorectal cancer. Cancer Res 2005;65(18):8072-8.

16. Samowitz WS, Slattery ML. Microsatellite instability in colorectal adenomas. Gastroenterology 1997;112(5):1515-9.

17. Samowitz WS, Slattery ML, Kerber RA. Microsatellite instability in human colonic cancer is not a useful clinical indicator of familial colorectal cancer. Gastroenterology 1995;109(6):1765-71.

18. Samowitz WS, Slattery ML. Regional reproducibility of microsatellite instability in sporadic colorectal cancer. Genes Chromosomes Cancer 1999;26(2):106-14.

19. Boland CR, Thibodeau SN, Hamilton SR, *et al.* A National Cancer Institute Workshop on Microsatellite Instability for cancer detection and familial predisposition: development of international criteria for the determination of microsatellite instability in colorectal cancer. Cancer Res 1998;58(22):5248-57.

20. Benlloch S, Paya A, Alenda C, *et al.* Detection of BRAF V600E mutation in colorectal cancer: comparison of automatic sequencing and real-time chemistry methodology. J Mol Diagn 2006;8(5):540-3.

21. Ogino S, Meyerhardt JA, Cantor M, *et al.* Molecular alterations in tumors and response to combination chemotherapy with gefitinib for advanced colorectal cancer. Clin Cancer Res 2005;11(18):6650-6.

22. Ogino S, Kawasaki T, Kirkner GJ, *et al.* CpG island methylator phenotype-low (CIMP-low) in colorectal cancer: possible associations with male sex and KRAS mutations. J Mol Diagn 2006;8(5):582-8.

23. Samowitz WS, Sweeney C, Herrick J, *et al.* Poor survival associated with the BRAF V600E mutation in microsatellite-stable colon cancers. Cancer Res 2005;65(14):6063-9.

24. Stewart CJ, Leung Y, Walsh MD, *et al.* KRAS mutations in ovarian low-grade endometrioid adenocarcinoma: association with concurrent endometriosis. Hum Pathol 2012;43(8):1177-83.

25. Alsop K, Mead L, Smith LD, *et al.* Low somatic K-ras mutation frequency in colorectal cancer diagnosed under the age of 45 years. Eur J Cancer 2006;42(10):1357-61.

26. Samowitz WS, Curtin K, Schaffer D, *et al.* Relationship of Ki-ras mutations in colon cancers to tumor location, stage, and survival: a population-based study. Cancer Epidemiol Biomarkers Prev 2000;9(11):1193-7.

27. Rosty C, Buchanan DD, Walsh MD, *et al.* Phenotype and polyp landscape in serrated polyposis syndrome: a series of 100 patients from genetics clinics. Am J Surg Pathol 2012;36(6):876-82.

28. Ogino S, Kawasaki T, Brahmandam M, *et al.* Sensitive sequencing method for KRAS mutation detection by Pyrosequencing. J Mol Diagn 2005;7(3):413-21.

29. Imamura Y, Lochhead P, Yamauchi M, *et al.* Analyses of clinicopathological, molecular, and prognostic associations of KRAS codon 61 and codon 146 mutations in colorectal cancer: cohort study and literature review. Mol Cancer 2014;13:135.
